# Post-transcriptional Regulation of HTLV Gene Expression: Rex to the Rescue

**DOI:** 10.3389/fmicb.2019.01958

**Published:** 2019-08-22

**Authors:** Donna M. D’Agostino, Ilaria Cavallari, Maria Grazia Romanelli, Vincenzo Ciminale

**Affiliations:** ^1^Department of Biomedical Sciences, University of Padova, Padua, Italy; ^2^Istituto Oncologico Veneto IOV – IRCCS, Padua, Italy; ^3^Section of Biology and Genetics, Department of Neurosciences, Biomedicine and Movement Sciences, University of Verona, Verona, Italy; ^4^Department of Surgery, Oncology and Gastroenterology, University of Padova, Padua, Italy

**Keywords:** HTLV-1, HTLV-2, splicing, RNA export, Rex

## Abstract

Human T-lymphotropic virus type 1 (HTLV-1) and other members of the Deltaretrovirus genus code for a regulatory protein named Rex that binds to the Rex-responsive element present on viral mRNAs. Rex rescues viral mRNAs from complete splicing or degradation and guides them to the cytoplasm for translation. The activity of Rex is essential for expression of viral transcripts coding for the virion components and thus represents a potential target for virus eradication. We present an overview of the functional properties of the HTLV-1 and HTLV-2 Rex proteins (Rex-1 and Rex-2), outline mechanisms controlling Rex function, and discuss similarities and differences in the sequences of Rex coded by HTLV-1, -2, -3, and -4 that may influence their molecular anatomy and functional properties.

## Introduction

Human T-lymphotropic virus type 1 (HTLV-1) infects approximately 10 million persons worldwide (Willems et al., 2017). HTLV-1 is the causative agent of adult T-cell leukemia/lymphoma (ATLL), tropical spastic paraparesis/HTLV-1-associated myelopathy (TSP/HAM) and several inflammatory diseases (Futsch et al., 2017). HTLV-1 is classified in seven molecular subtypes, named a, b, c, d, e, f, and g, with characteristic geographic distributions in several endemic regions (Gessain and Cassar, 2012). The closely related virus HTLV-2 circulates as two major subtypes, named a and b, mainly in indigenous populations of South America and western and central Africa, and in injection drug users (Roucoux and Murphy, 2004). Although the pathogenic spectrum of HTLV-2 is not clearly defined (Ciminale et al., 2014), infection with this virus may be associated with neurological disease (Araujo and Hall, 2004) and appears to significantly increase all-cause and cancer-related mortality (Biswas et al., 2010). Two other HTLVs, named HTLV-3 (Calattini et al., 2005; Wolfe et al., 2005) and HTLV-4 (Switzer et al., 2009), were identified in individuals living in the rainforests of Cameroon and are of unknown pathogenicity (Mahieux and Gessain, 2011). The HTLVs are classified in the Deltaretrovirus genus, which also includes the closely related simian T-lymphotropic viruses and bovine leukemia virus. Deltaretroviruses are considered to be “complex” retroviruses, as they produce regulatory and accessory proteins and exhibit 2-phase expression of alternatively spliced mRNAs (Cullen, 1991; Cavallari et al., 2011).

**FIGURE 1:**
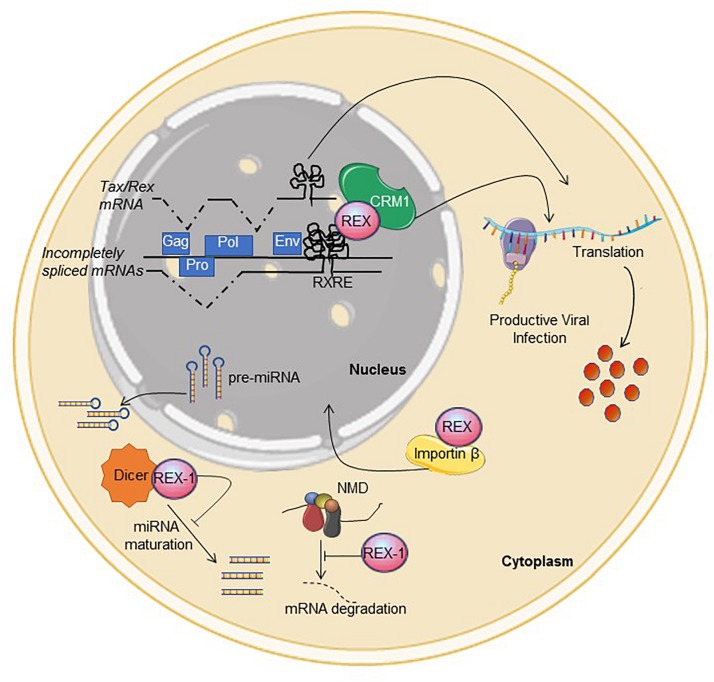
Functional activities described for Rex. See text for description. Interference with NMD and Dicer were described for Rex-1. The figure was made using SMART images (https://smart.servier.com).

The replication cycle of the HTLVs (and all Deltaretroviruses) is controlled by the viral regulatory proteins Tax and Rex, which are coded in open reading frames (ORFs) named x-IV and x-III, respectively, located on the plus-strand of the proviral genome. Tax increases transcription from the 5′LTR promoter as well as the promoters of many cellular genes, and plays a key role in viral replication and cell transformation (Romanelli et al., 2013). As illustrated in Figure 1, Rex regulates viral mRNA expression at the post-transcriptional level by interacting with a complex stem-loop RNA structure termed the Rex-responsive element (RXRE), present at the 3′ portion of all plus-strand viral transcripts. This interaction relieves the inhibitory effects of the RXRE and of cis-acting repressive sequences (CRS) present in incompletely spliced mRNAs, rescues these mRNAs from splicing or degradation, and allows their exit from the nucleus through a pathway mediated by the cellular export factor CRM1 (also referred to as exportin 1/XPO1) (Younis and Green, 2005).

The RXRE is located in the 3′LTR, with RXRE-1 mapping to a short portion of U3 and most of R (Toyoshima et al., 1990) and RXRE-2 including the entire R segment and a small portion of U5 (Kim et al., 1991). Its stem-loop structure brings the polyadenylation signal close to the polyadenylation site (Bar-Shira et al., 1991), ensuring efficient polyadenylation of viral mRNAs. This position suggests that all transcripts, including multiply spliced species, may have some degree of Rex-responsiveness; this was confirmed for constructs expressing HTLV-1 spliced mRNAs as intronless cDNAs (D’Agostino et al., 1999; Bai et al., 2012). An incomplete RXRE (R for RXRE-1 and R-U5 for RXRE-2) is also present at the 5′ end of the unspliced transcript.

In addition to CRS, introns and the RXRE, HTLV transcripts contain multiple stop codons, two ribosomal frameshifting signals and multiple splice acceptors that could be recognized by the non-sense-mediated decay (NMD) machinery. A study of HTLV-1-infected cells provided evidence that Rex contributes to suppress NMD of viral and cellular transcripts through a mechanism that does not involve interaction with the RXRE (Nakano et al., 2013). Results of *in vitro* experiments suggest that Rex-1 may also interfere with the activity of Dicer (Abe et al., 2010), a key component of the siRNA- and microRNA processing machinery. Its interactions with NMD and Dicer-dependent pathways suggest that Rex may have broad effects on cellular RNA processing and expression.

Studies of Rex and its HIV homolog Rev have contributed substantially to our knowledge of RNA processing as well as nucleo-cytoplasmic shuttling of proteins and RNA (Shida, 2012; Nakano and Watanabe, 2016; Rekosh and Hammarskjold, 2018). The following sections provide a brief description of Rex coded by HTLV-1 and HTLV-2, referred to as Rex-1 and Rex-2, respectively, and point out mechanisms that control Rex’s activity. We also comment on similarities and differences in the sequences of Rex-1, Rex-2, and Rex proteins coded by HTLV-3 and HTLV-4, whose activities have not been studied to date, and highlight aspects of Rex function that remain to be understood.

## Functional Domains in Rex-1 and Rex-2

**FIGURE 2:**
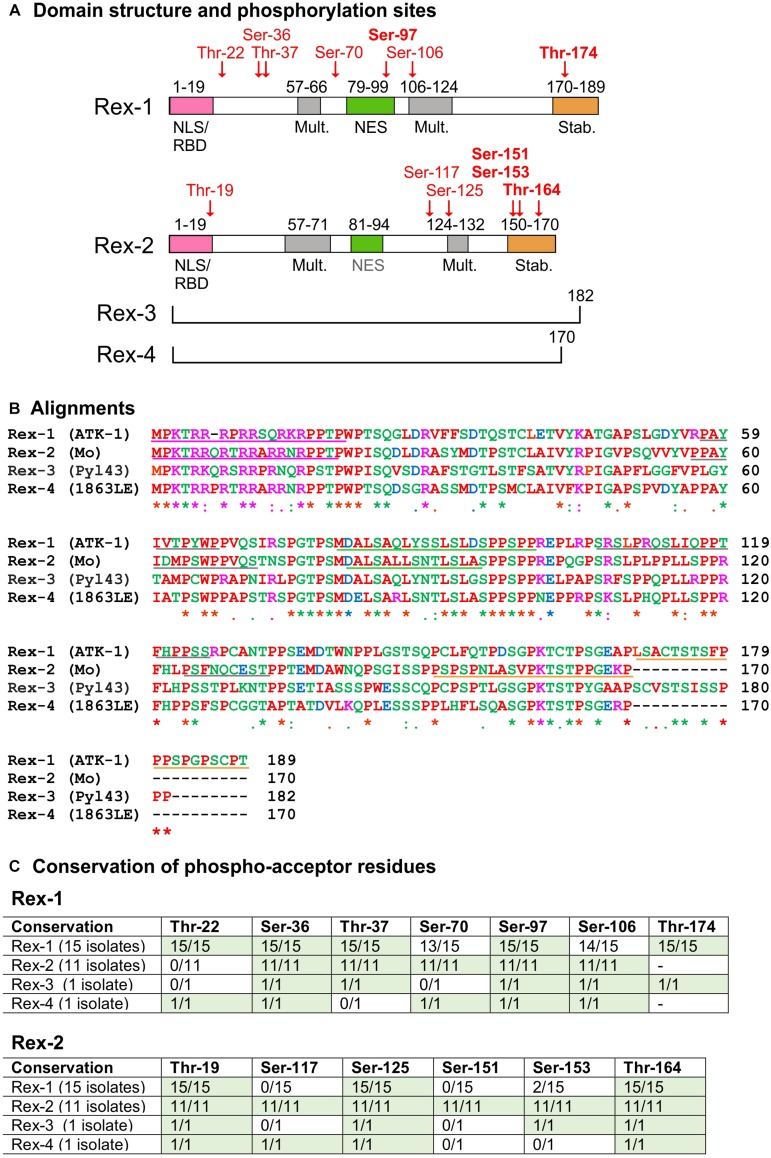
Domain structure and sequence comparison of HTLV Rex proteins. **(A)** shows the functional domain structure of Rex-1 and Rex-2. Positions of phosphoacceptor sites identified in Rex-1 (ATK-1) (Kesic et al., 2009a) and Rex-2 (Mo) (Kesic et al., 2009b) are indicated in red type. Diagrams are modified from Kesic et al. (2009a, b). **(B)** shows a multiple sequence alignment of Rex proteins obtained with Clustal Omega (http://www.ebi.ac.uk/Tools/msa/clustalo/). Amino acids are labeled in different colors according to their biochemical properties (red, small/hydrophobic; blue, acidic; magenta, basic; green, hydroxyl/sulfhydryl/amine/Glycine). Asterisks indicate single conserved residues; and periods indicate residues with similar properties. GenBank Accession IDs: HTLV-1 (ATK-1): J02029.1; HTLV-2 (Mo): M10060.1; HTLV-3 (Pyl 43): DQ462191.1; HTLV-4 (1863LE): EF488483.1 (see also Supplementary Figure 1). Tables in **(C)** show conservation of the indicated serines and threonines in Rex-1 and Rex-2 proteins coded by the panel of 28 viral isolates listed in Supplementary Table 1.

Almost all of the information on Rex was gathered from studies of Rex-1 coded by subtype-a isolate ATK-1 and Rex-2 coded by subtype-a isolate Mo. Rex-1 (ATK-1) and Rex-2 (Mo) contain 189- and 170 amino acids, respectively, and four main functional domains (Figure 2A; Younis and Green, 2005; Shida, 2012). An amino-terminal, arginine-rich nuclear/nucleolar localization signal (NLS) targets Rex to the nucleus through binding to importin β. This sequence also functions as an RNA binding domain (RBD) that mediates binding to the RXRE. A centrally positioned nuclear export sequence (NES) mediates binding of Rex to CRM1. The NES is flanked by two regions required for the formation of Rex multimers, a process that is facilitated by interaction with CRM1. A phosphorylation-regulated carboxy-terminal domain enhances Rex’s stability and function (Kesic et al., 2009a, b; Xie et al., 2009).

Figure 2B shows a CLUSTAL alignment of Rex-1 (ATK-1), Rex-2 (Mo) and the Rex-3 and Rex-4 proteins coded by 2 full-length HTLV-3 and HTLV-4 isolates (GenBank nos. listed in Supplementary Table 1). Overall, the NLS/RBD and NES show higher sequence identity compared to the multimerization- and stability domains. However, 9 of the 21 residues comprising Rex-2’s stability domain show perfect identity among the Rex proteins, suggesting a conserved functional role for this region. Percent-identity calculations showed that Rex-1 (ATK-1) is more similar to Rex-2 (Mo) than to Rex-3 or Rex-4, while Rex-2 (Mo) is most similar to Rex-4 (see Supplementary Table 2).

Rex-1 proteins coded by isolates of subtypes a, b, and c also showed some variation, especially between subtype-a and subtype-c ORFs (86.77–88.36% identity, see Supplementary Table 3). One subtype-a isolate, from an ATL patient in Iran, codes for Rex with 20 additional carboxy-terminal amino acids, a feature that might influence its stability domain (see Supplementary Figure 1). Alignments of 11 Rex-2 ORFs (5 subtype-a and 6 subtype-b) revealed 93.53–95.88% identity between the two subtypes (Supplementary Table 4), with distinct “signatures” of amino acids at positions 104, 105, 123, 126, and 136 (see arrows in Supplementary Figure 1). Interestingly, Rex-2 (Mo) was not the most common subtype-a sequence.

## Control of Rex Function by Phosphorylation

Early studies of Rex-1 showed that it migrates as a 27-kDa band in SDS-PAGE and is phosphorylated on multiple serines and threonines (Adachi et al., 1990, 1992). Mass spectrometry analysis of Rex-1 (ATK-1) (Kesic et al., 2009a) identified phosphorylation of Thr-22, Ser-36, Thr-37, Ser-70, Ser-97, Ser-106, and Thr-174 (see Figure 2A). Assays with a Gag-RXRE reporter plasmid and Rex-1 (ATK-1) mutants carrying phosphoablative (alanine) and phosphomimetic (aspartic acid) substitutions of these residues indicated that phospho-Ser-97, located in the NES, and phosho-Thr-174, located in the stability domain, contribute substantially to Rex activity (Kesic et al., 2009a).

Five of the seven phosphoacceptor sites identified in Rex-1 (ATK-1) (i.e., Thr-22, Ser-36, Thr-37, Ser-97, and Thr-174; Figure 2C) are conserved across the 15 HTLV-1 isolates aligned in Supplementary Figure 1. It is noteworthy that Ser-36 and Ser-97 are also conserved in Rex-2, Rex-3, and Rex-4 (see Supplementary Figure 1).

The Rex-2 (Mo) ORF produces two isoforms of 24- and 26 kDa that differ in phosphorylation (Green et al., 1991). Mass spectrometry analysis (Kesic et al., 2009b) showed that p26-Rex-2 is phosphorylated on Thr-19, Ser-117, Ser-125, Ser-151, Ser-153, and Thr-164 (see Figure 2A), while p24Rex-2 is phosphorylated on Ser-117 and Thr-164. Functional assays on point mutants revealed an important role for phosphorylation on Ser-151, Ser-153, and Thr-164, with phospho-Ser-151 particularly important for Rex-2’s ability to accumulate in the nucleus, bind to the RXRE and exert full functional activity (Narayan et al., 2001, 2003; Kesic et al., 2009b). These studies also suggested that sequential phosphorylation of Thr-164 followed by Ser-151 and Ser-153 converts the protein from a closed to an open conformation that exposes the NLS/RBD (Kesic et al., 2009b). Residues equivalent to Thr-19, Ser-125, and Thr-164 are present in all of the Rex proteins aligned in Supplementary Figure 1 (Figure 2C). The position of Thr-164 in a conserved region known to be important for stability and activation of Rex-2 suggests that it might be functionally relevant in all Rex proteins.

The protein kinases responsible for Rex phosphorylation have not been experimentally identified. Analysis of the Rex-1 (ATK-1) and Rex-2 (Mo) ORFs with the NetPhos and ScanSite prediction tools yielded one or more candidate kinases for most of the phosphoacceptor sites identified by mass spectrometry (Kesic et al., 2009a, b) as well as many other potential phosphoacceptor sites and kinases (Supplementary Table 5). Although the NetPhos and ScanSite predictions in general showed little agreement, both algorithms identified kinases potentially targeting Ser-97 in Rex-1 and the equivalent serine in Rex-2, Rex-3, and Rex-4 (Supplementary Table 5). Its conservation and position within the NES suggests that Ser-97 phosphorylation might influence Rex’s interaction with CRM1 and thereby modulate the nuclear export of cargo RNA.

Most of the studies of Rex phosphorylation have been carried out in cell lines of non-lymphoid derivation (e.g., 293T, Cos, HeLa) transfected with Rex expression plasmids. As the expression of protein kinases can be highly cell-type specific, it will be important to study the protein in cells that are natural targets of HTLV infection *in vivo* – predominantly CD4 ^+^ T-cells for HTLV-1 (Richardson et al., 1990; Melamed et al., 2015) and CD8 ^+^ T-cells for HTLV-2 (Ijichi et al., 1992; Melamed et al., 2014). The identification of Rex’s functionally relevant phospho-acceptor sites in these cells may have clinical implications, as inhibitors for some of the kinases predicted by NetPhos and ScanSite are already used in cancer therapy (Roskoski, 2019).

## Truncated Rex Isoforms

**FIGURE 3:**
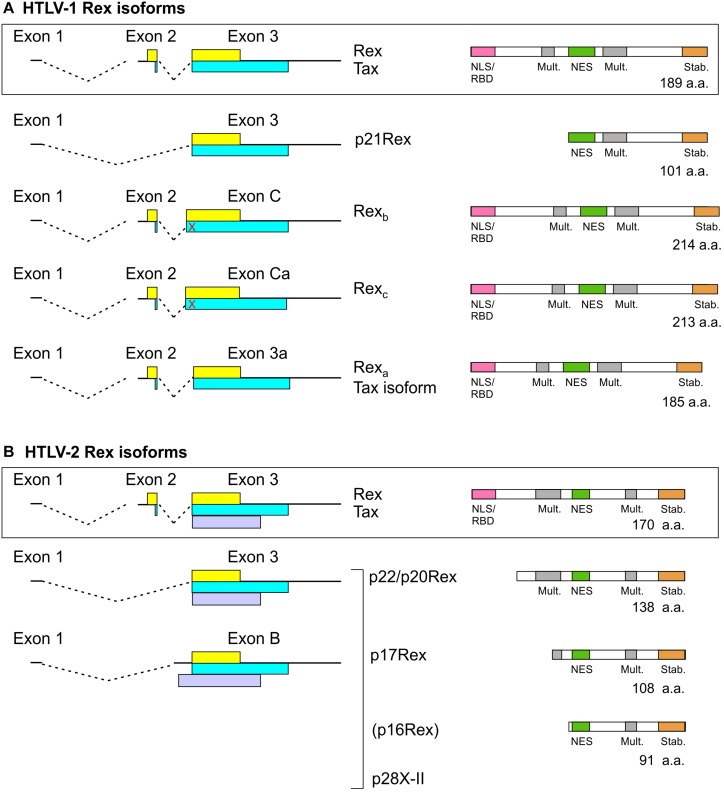
Rex isoforms coded by HTLV-1 **(A)** and HTLV-2 **(B)**. mRNAs coding for Tax/Rex and Rex isoforms are indicated on the left. The Tax and Rex ORFs are indicated by yellow and blue boxes, respectively, and the HTLV-2 p28 (x-II) ORF is indicated by a light purple box. The domain structures of Rex isoforms are indicated on the right. The red X indicates a stop codon upstream of the exon 3 splice acceptor that is in frame with the Tax ORF. p16Rex is a very low-abundance truncated Rex-2 isoform (Ciminale et al., 1995).

As depicted in Figure 3, Tax and Rex are expressed from a doubly spliced, bicistronic mRNA containing exons 1, 2, and 3. In addition to mRNA 1-2-3, HTLV-1, and HTLV-2 also produce transcripts that code for truncated forms of Rex. In HTLV-1, an mRNA that contains exon 1 linked to exon 3 (Orita et al., 1991) produces p21Rex, whose initiator methionine corresponds to methionine 79 of full-length Rex-1. p21Rex contains the NES, second multimerization domain segment and C-terminal stability domain of Rex-1, but lacks the N-terminal RBD/NLS (Figure 3A). While two studies indicated that p21Rex does not alter the function of full-length Rex-1 (Ciminale et al., 1997; Bai et al., 2012), another indicated an inhibitory effect (Heger et al., 1999), and a fourth study indicated that p21Rex, similar to full-length Rex, stabilizes the unspliced transcript through NMD inhibition (Nakano et al., 2013). Met-79 is present in all of the Rex ORFs examined in Supplementary Figure 1, so all of these viruses should be able to express p21Rex.

In HTLV-2, spliced mRNAs 1-3 and 1-B code for truncated Rex-2 proteins named p22/p20Rex and p17Rex (Ciminale et al., 1997; Figure 3B). These mRNAs also contain the x-II ORF, which codes for the regulatory protein p28 (Ciminale et al., 1995). The p22/20Rex isoforms initiate at Met-33 of full-length Rex-2 and contain both segments of the multimerization domain, the NES and the stability domain, while p17Rex starts at Met-63. Functional analyses of p22/p20 showed that they sequester full-length Rex-2 in the nucleus and interfere with its ability to activate expression of a RXRE-dependent RNA reporter. This effect was attributed to changes in Rex-2 phosphorylation induced by the truncated proteins rather than through the formation of inactive heteromultimers (Ciminale et al., 1997).

While Met-33 is not present in any of the 15 Rex-1 proteins or in Rex-3 shown in Supplementary Figure 1, it is present in 10 out of 11 Rex-2 proteins and in Rex-4. It is noteworthy that the Rex-1 ORFs coded by 2 Australian isolates examined in Supplementary Figure 1 contain a methionine seven codons downstream the position aligning with Met-33. These isolates, as well as HTLV-4, thus have the potential to produce proteins similar to p22/p20Rex and p21Rex.

## Functional Rex Isoforms

A search for novel monocistronic HTLV-1 transcripts coding for only Tax or Rex revealed the production of mRNAs that contain exons 1 and 2 linked to splice acceptors (SA) located upstream (C, Ca) or downstream (3a) of the canonical exon 3 SA; the use of these SA results in insertion or deletion of amino acids in the Rex ORF just after the NLS/RBD (Figure 3A; Rende et al., 2015). Transcripts 1-2-C and 1-2-Ca code for longer Rex-1 isoforms named Rex_b_ (214 a.a.) and Rex_c_ (213 a.a.), respectively, but do not produce Tax, and mRNA 1-2-3a produces a 185-residue Rex-1 isoform named Rex_a_ and a Tax isoform lacking four amino-proximal amino acids. Results of functional assays showed that all three Rex isoforms are functionally active. However, Rex_b_ and Rex_c_ are mainly cytoplasmic, suggesting that their extra amino acids affect the NLS and/or NES (Rende et al., 2015). It will be interesting to compare the expression of the alternatively spliced Tax/Rex and Rex isoform mRNAs in the context of asymptomatic infection and HTLV-1-associated pathologies.

The possibility that the other HTLVs produce analogous Rex isoforms remains to be investigated. SAs C, Ca and 3a are present in all 15 HTLV-1 isolates listed in Supplementary Table 1. However, six of the isolates, including the prototype ATK-1, contain a stop codon between SAs C and 3, and are thus predicted to produce Rex_a_ but not Rex_b_ or Rex_c_. Among the HTLV-2, HTLV-3, and HTLV-4 isolates in Supplementary Table 1, all but one are likewise predicted to produce a Rex_a_-like protein but not Rex_b_ or Rex_c_ due to the absence of the corresponding SAs and presence of one or more stop codons. HTLV-2b isolate Gu lacks all three alternative SAs and contains stop codons, and thus should not produce any of the extra Rex isoforms.

## The Two-Phase Model of HTLV Expression and Viral Latency

The first investigation of HTLV-1 mRNA expression kinetics, performed by transfecting a full-length HTLV-1 provirus and northern blotting, revealed early (Rex-independent) expression of multiply spliced mRNA and late (Rex-dependent) accumulation of singly spliced and unspliced mRNA (Hidaka et al., 1988). This two-phase model was supported and refined by time-course studies that employed quantitative RT-PCR with splice-site specific primers to detect individual alternatively spliced transcripts. Experiments performed in 293T cells transfected with an HTLV-1 molecular clone confirmed early accumulation of the Tax/Rex mRNA followed by a steady increase in the unspliced transcript and singly spliced env mRNA, but did not indicate Rex-dependence of other alternatively spliced species (Li et al., 2009, 2012). In partial contrast with these results, analyses of transfected and infected cell lines and cells from infected patients revealed that some of the alternatively spliced transcripts coding for accessory/regulatory proteins accumulate during the late phase together with the unspliced Gag-Pro-Pol and singly spliced Env mRNAs, indicating their Rex-dependence (Rende et al., 2011; Cavallari et al., 2016). All of the late mRNAs contain a 75-nucleotide 3′ CRS located between the splice acceptors for exons C and 3 that is absent from the Rex-independent mRNAs (Cavallari et al., 2016). These experiments also showed that mitosis partially overcomes the Rex-dependence of some transcripts, suggesting that Rex function is critical in the context of resting or slowly dividing cells (Cavallari et al., 2016). The 2-phase timecourse of mRNA expression was also evident in studies demonstrating the ability of Rex and p21Rex to block NMD (Nakano et al., 2013).

Mathematical modeling of HTLV-1 expression indicated the requirement for a delay in Rex function compared with Tax in order to support the 2-phase kinetics observed experimentally (Corradin et al., 2010). However, it is difficult to explain how Rex function might be delayed compared to Tax, given their co-expression by mRNA 1-2-3 (an early transcript). Possible contributing factors include (i) the shorter half-life of Tax and progressive accumulation of Rex, which is relatively stable (Rende et al., 2011); (ii) the temporal pattern of expression of potential inhibitors of Rex (e.g., p21Rex mRNA 1-3, expressed early); and (iii) late-phase accumulation of the monocistronic mRNAs coding for functional Rex isoforms Rex_b_ and Rex_c_ but not Tax.

**FIGURE 4:**
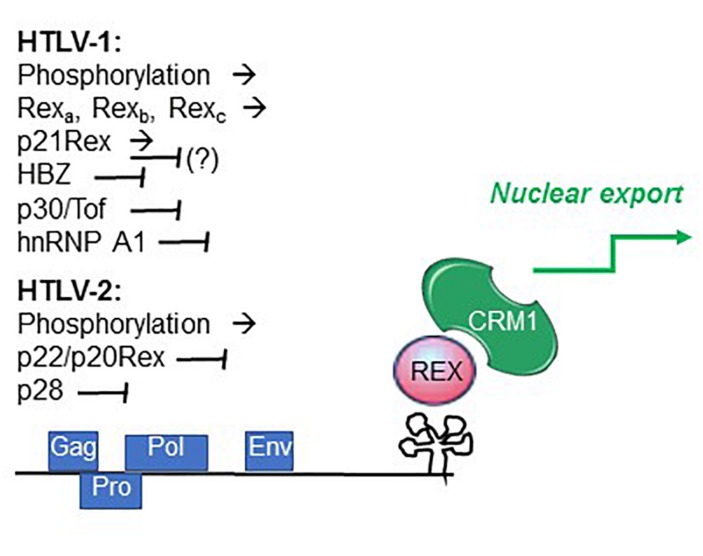
Factors controlling Rex function. Summarized are factors that promote (→) and interfere with (⊣) Rex function, as described in the text.

It is also possible that changes in the availability of cellular factors influence the relative activity of the 2 proteins during the expression cycle. An example is heterogenous nuclear ribonucleoprotein A1 (hnRNP A1), an important regulator of RNA processing that was shown to interfere with Rex function by competitively binding to the RXRE (Duc Dodon et al., 2002) (Figure 4).

Recent studies indicated that individual HTLV-1-infected cells undergo alternating bursts of viral gene expression and latency (Billman et al., 2017; Mahgoub et al., 2018). Shut-down of viral gene expression following the burst phase may involve the activity of the late-phase protein p30Tof, a nuclear/nucleolar product of the x-II ORF (Ciminale et al., 1992; Koralnik et al., 1993). In addition to important transcriptional effects, p30Tof blocks the Tax/Rex mRNA in the nucleus (Nicot et al., 2004), a property shared by the HTLV-2 x-II ORF protein p28 (Anupam et al., 2013). p30Tof also binds directly to Rex-1, resulting in interference with RXRE binding (Sinha-Datta et al., 2007) and failure to export RXRE-containing RNA cargo (Baydoun et al., 2007). Analyses of the intranuclear trafficking of Rex-1 showed that it normally encounters CRM1 within the nucleoli, and then travels as Rex-CRM1 complexes to nucleoplasmic foci, where it binds to RXRE-containing mRNA that will eventually be exported through the nuclear pore complex. p30/Tof blocks Rex-CRM1 complexes in the nucleoli, thus precluding their interaction with RNA cargo and blocking the RNA export leg (Baydoun et al., 2007). HBZ, a multifunctional regulatory protein coded on the minus strand of HTLV-1 (Ma et al., 2016), was also shown to interfere with Rex function (Philip et al., 2014) (Figure 4).

Studies of HTLV-2 expression kinetics indicated a similar 2-phase pattern of mRNA production (Bender et al., 2012). The observed late-phase expression of abundant levels of mRNA 1-3, coding for p22/20Rex and p28, suggests that these proteins may engage a negative feedback loop in the late phase through p22/p20Rex-mediated interference with Rex function and through p28-mediated retention of the Tax/Rex mRNA in the nucleus (Younis et al., 2004), thus favoring a shut-down of productive infection (Bender et al., 2012). The influence of the HTLV-2 minus-strand protein APH-2 on Rex function remains to be investigated.

## Conclusion and Perspectives

Comparisons of the biological properties of wild-type and Rex-defective HTLV-1 molecular clones indicated that while Rex is not essential for *in vitro* immortalization of cultured T-cells (a hallmark of HTLV-1 and HTLV-2), it is required for establishment of persistent infection in a rabbit model (Ye et al., 2003). This is an important finding, as persistent viral replication is considered to be a key factor that drives the inflammatory response to HTLV-1, with risk for developing TSP/HAM, and ensures the generation of a vast population of infected cells at risk for neoplastic transformation (Bangham, 2018).

The discovery of Rex-1 as an essential factor for expression of Gag/Pro/Pol RNA (Inoue et al., 1986) opened up a research field that yielded a wealth of information on the mechanisms regulating retroviral gene expression as well as cellular mRNA processing pathways. Further studies of Rex are needed in order to understand how its activity may be fine-tuned through phosphorylation and interactions with alternative Rex isoforms, other HTLV regulatory proteins, and cellular factors involved in mRNA processing, export and translation (Figure 4). These control points could be of key importance to turn on and off Rex function during the early/late phases the kinetics of expression of viral genes as well as in the bursts of expression revealed by the more recent single-cell analysis. It will be critical to focus future investigations of Rex-controlled HTLV gene expression on the natural cell targets of the virus, and to determine whether Rex phosphorylation, function and the pattern of splicing of viral transcripts change over time in infected individuals and, in the case of HTLV-1, are associated with development of disease. Answers to these questions could also pave the way to the development of novel therapeutic strategies to eradicate HTLV infection.

## Author Contributions

All authors worked together to prepare the manuscript. DD performed the sequence alignments and prepared the Supplementary  Material.

## Conflict of Interest Statement

The authors declare that the research was conducted in the absence of any commercial or financial relationships that could be construed as a potential conflict of interest.
