# Supplementary material for: Post-transcriptional Regulation of HTLV Gene Expression: Rex to the Rescue
Source: Front Microbiol. 2019 Aug 22;10:1958. doi: 10.3389/fmicb.2019.01958 (PMC6714889; doi:10.3389/fmicb.2019.01958)
Supplement: Supplementary file 1 [file Data_Sheet_1.PDF]

Supplemental Table 1. HTLV isolates whose Rex ORFs were analyzed for sequence conservation.

Information on subtype, isolate name, and geographic location were obtained from the GenBank record, from publications indicated therein, and, for some HTLV-1 isolates, from the HTLV-1 Molecular Epidemiology Database (http://htlv1db.bahia.fiocruz.br/). * isolate name or ** geographical origin not indicated in GenBank record. The potential to code for alternative Rex isoforms (columns 4 and 5) was evaluated by manual examination of the sequences.

GenBank Accession #	Virus/subtype (isolate name)	Geographic origin	Absence/presence of splice acceptors C/Ca/3a; stop codon(s) between exons C and 3	Predicted alternative Rex isoforms
J02029.1	HTLV-1/a (ATK-1)	Japan	+/+/+; 1 stop codon	Rex-3a
L36905.1	HTLV-1/a (*)	France	+/+/+	Rex-C, Rex-Ca, Rex-3a
U19949.1	HTLV-1/a (ATL-YS)	China	+/+/+; 1 stop codon	Rex-3a
D13784.1	HTLV-1/a (*)	Caribbean	+/+/+	Rex-C, Rex-Ca, Rex-3a
AF042071.1	HTLV-1/a (RKI3-Ger)	Germany	+/+/+	Rex-C, Rex-Ca, Rex-3a
AF259264.1	HTLV-1/a (WHP)	China	+/+/+	Rex-C, Rex-Ca, Rex-3a
AY563953.1	HTLV-1/a (*)	Brazil	+/+/+	Rex-C, Rex-Ca, Rex-3a
AY563954.1	HTLV-1/a (BRRP438)	Brazil	+/+/+	Rex-C, Rex-Ca, Rex-3a
HQ606137.1	HTLV-1/a (1066/05)	Canada	+/+/+	Rex-C, Rex-Ca, Rex-3a
HQ606138.1	HTLV-1/a (1067/05)	Canada	+/+/+	Rex-C, Rex-Ca, Rex-3a
MH399769.1	HTLV-1/a (MhD ATL5)	Iran	+/+/+	Rex-C, Rex-Ca, Rex-3a
JX507077.1	HTLV-1/b (SF26)	Brazil	+/+/+; 1 stop codon	Rex-3a
L02534.1	HTLV-1/c (MEL5)	Solomon Islands	+/+/+; 1 stop codon	Rex-3a
JX891478.1	HTLV-1/c (Aus-GM)	Australia	+/+/+; 1 stop codon	Rex-3a
JX891479.1	HTLV-1/c (Aus-NR)	Australia	+/+/+; 1 stop codon	Rex-3a
M10060.1	HTLV-2/a (Mo)	**	-/-/+; 2 stop codons	Rex-3a
AF139382.1	HTLV-2/a (SP-WV)	Brazil	-/-/+; 2 stop codons	Rex-3a
AF326583.1	HTLV-2/a (RP329)	Brazil	-/-/+; 2 stop codons	Rex-3a
AF326584.1	HTLV-2/a (k96)	Brazil	-/-/+; 2 stop codons	Rex-3a
AF412314.1	HTLV-2/a (*)	**	-/-/+; 2 stop codons	Rex-3a
L11456.1	HTLV-2/b/G12	Panama	-/-/+; 2 stop codons	Rex-3a
GU212854.1	HTLV-2/b (Pygmy 550602)	Cameroon	-/-/+; 1 stop codon	Rex-3a
X89270.1	HTLV-2/b/Gu	Italy	-/-/-; 2 stop codons	(-)
Y13051.1	HTLV-2/b (Gab)	Gabon	-/-/+; 2 stop codons	Rex-3a
AF074965.1	HTLV-2/b (G2)	Venezuela	-/-/+; 2 stop codons	Rex-3a
L20734.1	HTLV-2/b (NRA)	**	-/-/+; 1 stop codon	Rex-3a
DQ462191.1	HTLV-3 (Pyl 43)	Cameroon	-/-/+; 1 stop codon	Rex-3a
EF488483.1	HTLV-4 (1863LE)	Cameroon	-/-/+; 2 stop codons	Rex-3a

Supplemental Figure 1. Sequence alignments of Rex ORFs. Rex ORFs from the indicated virus isolates were analyzed with Clustal Omega (http://www.ebi.ac.uk/Tools/msa/clustalo/). Amino acids are labeled in different colors according to their biochemical properties (red: small/hydrophobic; blue: acidic; magenta: basic; green: hydroxyl/sulfhydryl/amine/Glycine). Asterisks indicate single conserved residues, periods indicate residues with similar properties, and blue arrows indicate 'signature' residues 104, 105, 123, 126 and 136 that distinguish HTLV-2a isolates from HTLV-2b isolates. GenBank accession nos. of Rex-1 (ATK-1) and Rex-2 (Mo) are in bold type.

```
DQ462191.1(3)       MPKTRKQRSRRPRNQRPSTPWPISQVSDRAFSTGTLSTFSATVYRPIGAPFLGGFVPLGY    60
JX507077.1(1-b)     MPKTRR-GPRRSQRKRPPTPWPTSQGLDRVLFSDTQSTCLETVYKATGAPSLEDYVRPAY    59
D13784.1(1-a)       MPKTRR-RPRRSQRKRPPTPWPTSQGLDRVFFSDTQSTCLETVYKATGAPSLGDYVRPAY    59
L36905.1(1-a)       MPKTRR-RPRRSQRKRPPTPWPTSQGLDRVFFSDTQSTCLETVYKATGAPSLGDYVRPAY    59
AY563953.1(1-a)     MPKTRR-RPRRSQRKRPPTPWPTSQGLDRVFFSDTQSTCLETVYKAIGAPSLGDYVRPAY    59
AY563954.1(1-a)     MPKTRR-RPRRSQRKRPPTPWPTSQGLDRVFFSDTQSTCLETVYKAIGAPSLGDYVRPAY    59
AF259264.1(1-a)     MPKTRR-RPRRSQRKRPPTPWPTSQGLDRVFFSDTQSTCLETVYKATGAPSLGDYVRPAY    59
AF042071.1(1-a)     MPKTRR-RPRRSQRKRPPTPWPTSQGLDRVFFSDTQSTCLETVYKATGAPSLGDYVRPAY    59
MH399769.1(1-a)     MPKTRR-RPRRSQRKRPPTPWPTSQGLDRVFFSDTQSTCLETVYKATGAPSLGDYVRPAY    59
J02029.1(1-a)       MPKTRR-RPRRSQRKRPPTPWPTSQGLDRVFFSDTQSTCLETVYKATGAPSLGDYVRPAY    59
U19949.1(1-a)       MPKTRR-RPRRSQRKRPPTPWPTSQGLDRVFFSDTQSTCLETVYKATGAPSLGDYVRPAY    59
HQ606137.1(1-a)     MPKTRR-RPRRSQRKRPPTPWPTSQGLDRVFFSDTQSTCLETVYKATGAPSLGDYVRPAY    59
HQ606138.1(1-a)     MPKTRR-RPRRSQRKRPPTPWPTSQGLDRVFFSDTQSTCLETVYKATGAPSLGDYVRPAY    59
L02534.1(1-c)       MPKTRR-GPRRSQRKRPPTPWPTSQGLDKVFFTDIQSTCLETVYKATGAPSLGDYVRPAY    59
JX891479.1(1-c)     MPKTRR-GPRRSQRKRPPTPWPTSQGLDKVFFTDIQSTCMETVYKATGAPSLGDYARPAY    59
JX891478.1(1-c)     MPKTRR-GPRRSQRKRPPTPWPTSQGLDKVFFTDIQSTCMETVYKATGAPSLGDYARPAY    59
EF488483.1(4)       MPKTRRPRTRRARRNRPPTPWPTSQDSGRASSMDTPSMCLAIVFKPIGAPSPVDYAPPAY    60
AF326584.1(2-a)     MPKTRRQRTRRARRNRPPTP--ISQDLDRASYMDTPSTCLAIVYRPIGVPSQVVYVPPAY    58
AF326583.1(2-a)     MPKTRRQRTRRARRNRPPTPWAISQDLDRASYMDTPSTCLAIVYRPIGVPSQVVYVPPAY    60
AF412314.1(2-a)     MPKTRRQRTRRARRNRPPTPWPISQDLDRASYMDTPSTCLAIVYRPIGVPSQVVYVPPAY    60
M10060.1(2-a)       MPKTRRQRTRRARRNRPPTPWPISQDLDRASYMDTPSTCLAIVYRPIGVPSQVVYVPPAY    60
AF139382.1(2-a)     MPKTRRQRTRRARRNRPPTPWPISQDLDRASYMDTPSTCLAIVYRPIGVPSQVVYVPPAY    60
X89270.1(2-b)       MPKTRRQRTRRARRNRPPTPWPIPKDSDRASYMDTPSTCLAIVYRPIGVPSQVVYVPPAY    60
GU212854.1(2-b)     MPKTRRQRTRRARRNRPPTPWPISQDLDRASYIDTPSTCLAIVYRPIGVPSQVVYVPPAY    60
Y13051.1(2-b)       MPKTRRQRTRRARRNRPPTPWPISQDSDRASYMDTPSTCLAIVYRPIGVPSQVVYVPPAY    60
AF074965.1(2-b)     MPKTRRQRTRRARRNRPPTPWPISQDSDRASYMDTPSTCLAIVYRPIGVPSQVVYVPPAY    60
L11456.1(2-b)       MPKTRRQRTRRARRNRPPTPWPISQDSDRASYMDTPSTCLAIVYRPIGVPSQVVYVPPAY    60
L20734.1(2-b)       MPKTRRQRTRRARRNRPPTPWPISQDSDRASYMDTPSTCLAIVYRPIGVPSQVVYVPPAY    60
                    *****:   ** :.:** **    :  .:.    .  *     *::  *.*    :.  .*

DQ462191.1(3)       TAMPCWPRAPNIRLPGTPSMDALSAQLYNTLSLGSPPSPPKELPAPSRFSPPQPLLRPPR    120
JX507077.1(1-b)     IVTPYWPPVQSIRSPGTPSMDALSAQLYSSLSLGSPPSPPREPLRPSRSLPRRPPIQPPT    119
D13784.1(1-a)       IVTPYWPPVQSIRSPGTPSMDALSAQLYSSLSLDSPPSPPREPLRPLRSLPRQSLIQPPT    119
L36905.1(1-a)       IVTPYWPPVQSIRSPGTPSMDALSAQLYSSLSLDSPPSPPREPLRPSRSLPRQSLIQPPT    119
AY563953.1(1-a)     IVTPYWPPVQSIRSPGTPSMDALSAQLYSSLSLDSPPSPPREPLRPSRSLPRQSLIQPPT    119
AY563954.1(1-a)     IVTPYWPPVQSIRSPGTPSMDALSAQLYSSLSLDSPPSPPREPLRPSRSLPRQSLIQPPT    119
AF259264.1(1-a)     IVTPYWPPVQSIRSPGTPSMDALSAQLYSSLSLDSPPSPPREPLRPSRSLPRQSLIQPPT    119
AF042071.1(1-a)     IVTPYWPPVQSIRSPGTPSMDALSAQLYSSLSLDSPPSPPREPLRPSRSLPRQSLIRPPT    119
MH399769.1(1-a)     IVTPYWPPVQSIRSPGTPSMDALSAQLYSSLSLDSPPSPPREPLRPSRSLPRQSLIQPPT    119
J02029.1(1-a)       IVTPYWPPVQSIRSPGTPSMDALSAQLYSSLSLDSPPSPPREPLRPSRSLPRQSLIQPPT    119
U19949.1(1-a)       IVTPYWPPVQSIRSPGTPSMDALSAQLYSSLSLDSPPSPPREPLRPSRSLPRQSLIQPPT    119
HQ606137.1(1-a)     IVTPYWPPVQSIRSPGTPSMDALSAQLYSSLSLDSPPSPPREPLRPSRSLPRQSLIQPPT    119
HQ606138.1(1-a)     IVTPYWPPVQSIRSPGTPSMDALSAQLYSSLSLDSPPSPPREPLRPSRSLPRQSLIQPPT    119
L02534.1(1-c)       IVTPYWPPVQSIRSPRTPSMDALSAQLYSSLSLGSPPSPPREPLKPSRSLPHRPLIQPPT    119
JX891479.1(1-c)     IVTPYWPPAQNIRSPGTPSMDALSAQLYNSLSLDSPPSPPREPLRPSRSLPHRPLIQPPT    119
JX891478.1(1-c)     IVTPYWPPAQNIRSPGTPSMDALSAQLYNSLSLDSPPSPPREPLRPSRSLPHRPLIQPPT    119
EF488483.1(4)       IATPSWPPAPSTRSPGTPSMDELSARLSNTLSLASPPSPPNEPPRPSKSLPHQPLLSPPR    120
AF326584.1(2-a)     IDMPSWPPVQSTNSPGTPSMDALSALLSNTLSLASPPSPPREPQGPSRSLPLPPLLSPPR    118
AF326583.1(2-a)     IDMPSWPPVQSTNSPGTPSMDALSALLSNTLSLASPPSPPREPQGPSRSLPLPPLLSPPR    120
AF412314.1(2-a)     IDMPSWPPVQSTNSPGTPSMDALSALLSNTLSLASPPSPPREPQGPSRSLPLPPLLSPPR    120
M10060.1(2-a)       IDMPSWPPVQSTNSPGTPSMDALSALLSNTLSLASPPSPPREPQGPSRSLPLPPLLSPPR    120
AF139382.1(2-a)     IDMPSWPPVQSTNSPGTPSMDALSALLSNTLSLASPPSPPREPQGPSRSLPLPPLLSPPR    120
X89270.1(2-b)       IDMPSWPPVQSTNSPGTPSMDALSALLSNTLSLASPPSPPREPPRPSRSLPLPPLLSPPR    120
GU212854.1(2-b)     IDMPSWPPVQSTSSPGTPSMDALSALLSNTLSLASPPSPPREPPRPSRSLPLPPLLSPPR    120
Y13051.1(2-b)       IDMPSWPPVQSTSSPGTPSMDTLSALLSNTLSLASPPSPPREPPRPSRSLPLPPLLSPPR    120
AF074965.1(2-b)     IDMPSWPPVQSTSSPGTPSMDALSALLSNTLSLASPPSPPREPPRPSRSLPLPPLLSPPR    120
L11456.1(2-b)       IDMPSWPPVQSTSSPGTPSMDALSALLSNTLSLASPPSPPREPPRPSRSLPLPPLLSPPR    120
L20734.1(2-b)       IDMPSWPPVQSTSSPGTPSMDALSALLSNTLSLASPPSPPREPPRPSRSLPLPPLLSPPR    120
                       * ** . .   * ***** *** * .:*** ******.* ↑↑ * :  *    : **
```

```
DQ462191.1(3)      FLHPSSTPLKNTPPSETIASSSPWESSCQPCPSPTLGSGPKTSTPYGAAPSCVSTSISSP   180
JX507077.1(1-b)    FHPPSSRPCANTPPSETDTWNPPLGSTSQPCLFQTPASGPRTCTPSGEAPLSACTSTSFP   179
D13784.1(1-a)      FHPPSSRPCANTPPSEMDTWNPPLGSTSQPCLFQTPDSGPKTCTPSGEAPLSACTSTSFP   179
L36905.1(1-a)      FHPPSSRPCANTPPSEMDTWNPPLGSTSQPCLSQTPDSGPKTCTPSGEAPLSACTSTSFP   179
AY563953.1(1-a)    FHPPSSRPCANTPPSEMDTWNPPLGSTSQPCLFQTPDSGPKTCTPSGEAPLSACTSTSFP   179
AY563954.1(1-a)    FHPPSSRPCANTPPSEMDTWNPPLGSTSQPCLFQTPDSGPKTCTPSGEAPLSACTSTSFP   179
AF259264.1(1-a)    FHPPSSRPCANTPPSEMDTWNPPLGSTSQPCLFQTPDSGPKTCTPFGEAPLSACTSTSFP   179
AF042071.1(1-a)    FHPPSSRPCANTPPSEMDTWNPPLGSTSQPCLFQTPDSGPKTCTPSGEAPLSACTSTSFP   179
MH399769.1(1-a)    FHPPSSRPCANTPPSEMDTWNPPLGSTSQPCLFQTPDSGPKTCTPSGEAPLSACTSTSFP   179
J02029.1(1-a)      FHPPSSRPCANTPPSEMDTWNPPLGSTSQPCLFQTPDSGPKTCTPSGEAPLSACTSTSFP   179
U19949.1(1-a)      FHPPSSRPCANTPPSEMDTWNPPLGSTSQPCLFQTPDSGPKTCTPSGEAPLSACTSTSFP   179
HQ606137.1(1-a)    FHPPSSRPCANTPPSEMDTWNPPLGSTSQPCLFQTPDSGPKTCTPSGEAPLSACTSTSFP   179
HQ606138.1(1-a)    FHPPSSRPCANTPPSEMDTWNPPLGSTSQPCLFQTPDSGPKTCTPSGEAPLSACTSTSFP   179
L02534.1(1-c)      FHPPSSRPYANTPPSEMGAWSPPLGSSSQACPSPTPASGPKTCTPSGEAPSSACTSISFP   179
JX891479.1(1-c)    FHPPSSRPYANTPPSEMGTWSPPLGSSSQACPFPTPASGPKTYTPSGEAPSSACTSISFP   179
JX891478.1(1-c)    FHPPSSRPYANTPPSEMDTWSPPLGSSSQACPFPTPASGPKTYTPSGEAPSSACTSISFP   179
EF488483.1(4)      FHPPSFSPCGGTAPTATDVLKQPLESSSPPLHFLSQASGPKTSTPSGERP----------   170
AF326584.1(2-a)    FHLPSFNQCESTPPTEMDAWNQPSGISSPPSPSLNLASVPKTSTPPGEKP----------   168
AF326583.1(2-a)    FHLPSFNQCESTPPTEMDAWNQPSGISSPPSPSLNLASVPKTSTPPGEKP----------   170
AF412314.1(2-a)    FHLPSFNQCESTPPTEMDAWNQPSGISSPPSPSPNLASVPKTSTPPGEKP----------   170
M10060.1(2-a)      FHLPSFNQCESTPPTEMDAWNQPSGISSPPSPSPNLASVPKTSTPPGEKP----------   170
AF139382.1(2-a)    FHLPSFNQCESTPPTEMDAWNQPSGISSPPSPSLNLASVPKTSTPPGEKP----------   170
X89270.1(2-b)      FHPPSSNQCESTPPIAMDAWNQPSGISSPPSPSPNLASVPKTSTPPGEKP----------   170
GU212854.1(2-b)    FHPPSSNQCENTPPIAMDAWNQPSGISSPPSPSLNLASVPKTSTPPGEKP----------   170
Y13051.1(2-b)      FHPPSSNQCESTPPIAMDAWNQPSGISSPPSPSLNLASVPKTSTPPGEKP----------   170
AF074965.1(2-b)    FHPPSSNQCESTPPTAMDAWNQPSGISSPPSPSLNLASVPKTSTPPGEKP----------   170
L11456.1(2-b)      FHPPSSNQCESTPPIAMDAWNQPSGISSPPSPSLNLASVPKTSTPPGEKP----------   170
L20734.1(2-b)      FHPPSSNQCESTPPIAMDAWNQPSGISSPPSPSLNLASVPKTSTPPGEKP----------   170
                   * ↑**↑    .* * ↑  . . *   :.      .  * *:* ** *  *

DQ462191.1(3)      PP----------------------------        182
JX507077.1(1-b)    PPSPGPSCPM--------------------        189
D13784.1(1-a)      PPSPGPSCPM--------------------        189
L36905.1(1-a)      PPSPGPSCPT--------------------        189
AY563953.1(1-a)    PPSPGPSCPT--------------------        189
AY563954.1(1-a)    PPSPGPSCPT--------------------        189
AF259264.1(1-a)    PPSPGPSCPT--------------------        189
AF042071.1(1-a)    PPSPGPSCPT--------------------        189
MH399769.1(1-a)    PPSPGPSCPTWFFATPASSGPSSPMSPTSE        209
J02029.1(1-a)      PPSPGPSCPT--------------------        189
U19949.1(1-a)      PPSPGPSCPT--------------------        189
HQ606137.1(1-a)    PPSPGPSCPT--------------------        189
HQ606138.1(1-a)    PPSPGPSCPT--------------------        189
L02534.1(1-c)      PPSPGPSCPR--------------------        189
JX891479.1(1-c)    PPSPGPSCPR--------------------        189
JX891478.1(1-c)    PPSPGPSCPR--------------------        189
EF488483.1(4)      ------------------------------        170
AF326584.1(2-a)    ------------------------------        168
AF326583.1(2-a)    ------------------------------        170
AF412314.1(2-a)    ------------------------------        170
M10060.1(2-a)      ------------------------------        170
AF139382.1(2-a)    ------------------------------        170
X89270.1(2-b)      ------------------------------        170
GU212854.1(2-b)    ------------------------------        170
Y13051.1(2-b)      ------------------------------        170
AF074965.1(2-b)    ------------------------------        170
L11456.1(2-b)      ------------------------------        170
L20734.1(2-b)      ------------------------------        170
```

Supplemental Table 2. Percent identity matrix for Rex-1 (ATK-1), Rex-2 (Mo), Rex-3 and Rex-4.

	ATK-1	Mo	Py143	1863LE
**Rex-1 (ATK-1)**	100.00	62.72	56.35	62.13
**Rex-2 (Mo)**	62.72	100.00	57.65	71.76
**Rex-3 (Py143)**	56.35	57.65	100.00	55.88
**Rex-4 (1863LE)**	62.13	71.76	55.88	100.00

Supplemental Table 3. Rex-1 Percent identity matrix. Rex ORFs coded by 15 HTLV-1 isolates were analyzed with Clustal Omega. Shown is the resulting percent identity matrix and a summary of results. Subtype-a isolates are in black, one subtype-b isolate is in red, and 3 subtype-c isolates are in green.

JX507077.1	100.00	94.18	93.65	93.65	93.65	93.65	93.65	94.18	94.18	94.18	94.18	94.18	87.83	86.24	86.77
D13784.1	94.18	100.00	98.41	98.41	98.41	98.41	98.41	98.94	98.94	98.94	98.94	98.94	87.30	86.77	87.30
L36905.1	93.65	98.41	100.00	98.94	98.94	98.94	98.94	99.47	99.47	99.47	99.47	99.47	88.36	86.77	87.30
AY563953.1	93.65	98.41	98.94	100.00	100.00	98.94	98.94	99.47	99.47	99.47	99.47	99.47	87.30	86.77	87.30
AY563954.1	93.65	98.41	98.94	100.00	100.00	98.94	98.94	99.47	99.47	99.47	99.47	99.47	87.30	86.77	87.30
AF259264.1	93.65	98.41	98.94	98.94	98.94	100.00	98.94	99.47	99.47	99.47	99.47	99.47	87.30	86.77	87.30
AF042071.1	93.65	98.41	98.94	98.94	98.94	98.94	100.00	99.47	99.47	99.47	99.47	99.47	87.30	86.77	87.30
MH399769.1	94.18	98.94	99.47	99.47	99.47	99.47	99.47	100.00	100.00	100.00	100.00	100.00	87.83	87.30	87.83
**J02029.1**	94.18	98.94	99.47	99.47	99.47	99.47	99.47	100.00	100.00	100.00	100.00	100.00	87.83	87.30	87.83
U19949.1	94.18	98.94	99.47	99.47	99.47	99.47	99.47	100.00	100.00	100.00	100.00	100.00	87.83	87.30	87.83
HQ606137.1	94.18	98.94	99.47	99.47	99.47	99.47	99.47	100.00	100.00	100.00	100.00	100.00	87.83	87.30	87.83
HQ606138.1	94.18	98.94	99.47	99.47	99.47	99.47	99.47	100.00	100.00	100.00	100.00	100.00	87.83	87.30	87.83
L02534.1	87.83	87.30	88.36	87.30	87.30	87.30	87.30	87.83	87.83	87.83	87.83	87.83	100.00	94.18	93.65
JX891479.1	86.24	86.77	86.77	86.77	86.77	86.77	86.77	87.30	87.30	87.30	87.30	87.30	94.18	100.00	99.47
JX891478.1	86.77	87.30	87.30	87.30	87.30	87.30	87.30	87.83	87.83	87.83	87.83	87.83	93.65	99.47	100.00

Summary

11 subtype-a isolates: 98.41-100% identity
3 subtype-c isolates: 93.65-99.47% identity
11 subtype-a vs. 1 subtype-b isolate: 93.65-94.18% identity
11 subtype-a vs. 3 subtype-c isolates: 86.77-88.36% identity
1 subtype-b vs. 3 subtype c isolates: 86.24-87.83% identity

Supplemental Table 4. Rex-2 Percent identity matrix. Rex ORFs coded by 11 HTLV-2 isolates were analyzed with Clustal Omega. Shown is the resulting percent identity matrix and a summary of results. Subtype-a isolates are in black and subtype-b isolates are in red.

AF326584.1	100.00	100.00	99.40	99.40	100.00	94.05	94.64	94.64	95.83	95.24	95.24
AF326583.1	100.00	100.00	98.82	98.82	99.41	93.53	94.12	94.12	95.29	94.71	94.71
AF412314.1	99.40	98.82	100.00	100.00	99.41	94.71	94.12	94.12	95.29	94.71	94.71
M10060.1	99.40	98.82	100.00	100.00	99.41	94.71	94.12	94.12	95.29	94.71	94.71
AF139382.1	100.00	99.41	99.41	99.41	100.00	94.12	94.71	94.71	95.88	95.29	95.29
X89270.1	94.05	93.53	94.71	94.71	94.12	100.00	95.88	97.06	97.06	97.65	97.65
GU212854.1	94.64	94.12	94.12	94.12	94.71	95.88	100.00	97.65	97.65	98.24	98.24
Y13051.1	94.64	94.12	94.12	94.12	94.71	97.06	97.65	100.00	98.82	99.41	99.41
AF074965.1	95.83	95.29	95.29	95.29	95.88	97.06	97.65	98.82	100.00	99.41	99.41
L11456.1	95.24	94.71	94.71	94.71	95.29	97.65	98.24	99.41	99.41	100.00	100.00
L20734.1	95.24	94.71	94.71	94.71	95.29	97.65	98.24	99.41	99.41	100.00	100.00

Summary

5 subtype-a isolates: 98.82-100% identity
6 subtype-b isolates: 95.88-100% identity
5 subtype-a vs. 6 subtype-b isolates: 93.53-95.88% identity

Supplemental Table 5. Protein kinase predictions for selected Serines and Threonines in Rex.

NetPhos 3.1 (http://www.cbs.dtu.dk/services/NetPhos/), Scansite 4 (https://scansite4.mit.edu/4.0/#home). Unsp., unspecified kinase. [ ][1] Numbered according to Rex-1; add +1 for corresponding residues in Rex-2, 3 and 4. [ ][2] Numbered according to Rex-2; subtract 1 for corresponding residues in Rex-1. (-), no kinases predicted using default cutoff score (>0.5, NetPhos 3.1) or medium stringency (ScanSite 4).

**Rex-1**	**NetPhos 3.1**	**ScanSite 4**
Thr-22	(-)	(-)
Ser-36	CK2	(-)
Thr-37	Unsp., CK2	(-)
Ser-70	PKC	NEK2
Ser-97	Unsp., CDK5, p38MAPK, GSK3	CDK1, CDK5, CDC2, ERK1, GSK3
Ser-106	Unsp., PKA, CDC2	(-)
Ser-174	(-)	(-)
[Thr-19][2]	Unsp., PKB, CDK5, GSK3	(-)
[Ser-125][2]	Unsp.	(-)
[Thr-164][2]	Unsp., CDK5	(-)

**Rex-2**	**NetPhos 3.1**	**ScanSite 4**
Thr-19	Unsp., PKB, CDK5, GSK3	ERK1
Ser-117	CDK5, p38MAPK, GSK3	ERK1
Ser-125	(-)	PDK1
Ser-151	Unsp., CDK5, GSK3, Cdc2, p38MAPK	ERK1, CDK1, GSK3
Ser-153	CDK5	(-)
Thr-164	Unsp., CDK5	(-)
[Ser-36][1]	Cdc2	(-)
[Thr-37][1]	PKC	(-)
[Ser-97][1]	Unsp., p38MAPK, GSK3, CDK5	CDK5, CDK1, Cdc2, ERK1
[Ser-106][1]	PKA, Cdc2	(-)

**Rex-3**	**NetPhos 3.1**	**ScanSite 4**
Thr-19	Unsp., CDK5	(-)
[Ser-36][1]	PKC, Cdc2	(-)
[Thr-37][1]	PKC	(-)
[Ser-97][1]	Unsp., p38MAPK, CDK5, GSK3	CDK5, CDK1, Cdc2, ERK1
[Ser-106][1]	(-)	(-)
Ser-125	(-)	(-)
Ser-153	P38MAPK, CDK5	(-)
Thr-164	Unsp., CDK5, Cdc2	(-)
[Thr-174][1]	PKC	GSK3, GSK3B

**Rex-4**	**NetPhos 3.1**	**ScanSite 4**
Thr-19	Unsp., PKB, CDK5, GSK3	(-)
[Thr-22][1]	(-)	GSK3B
[Ser-36][1]	Cdc2	(-)
[Ser-97][1]	Unsp., CDK5, GSK3, p38MAPK	ERK1
[Ser-106][1]	Unsp., PKA	(-)
Ser-117	CDK5, p38MAPK, GSK3	ERK1
Ser-125	(-)	PDK1
Thr-164	Unsp., CDK5	(-)
